# Intrathecal Baclofen for Hypertonia Secondary to Glutaric Aciduria Type I

**DOI:** 10.7759/cureus.8818

**Published:** 2020-06-25

**Authors:** Max Frenkel, Emily J Meyer, James A Stadler

**Affiliations:** 1 Neurological Surgery, University of Wisconsin School of Medicine and Public Health, Madison, USA

**Keywords:** glutaric aciduria type i, ga1, intrathecal baclofen, dystonia, spasticity, pediatric neurosurgery

## Abstract

Glutaric aciduria type I (GA1) is a rare organic aciduria characterized by basal ganglia dysfunction and severe dystonia and spasticity for which enteral baclofen is currently first-line therapy. Intrathecal baclofen (ITB) is a promising alternative, given the dose titratability and concentrated delivery of medication to therapeutic targets within the central nervous system. However, the response to ITB in patients with this rare condition has not been previously reported. We present a 15-year-old girl with GA1 and associated hypertonia refractory to extensive, multimodal adjuvant medical therapy including enteral baclofen. An ITB pump was implanted, and after an appropriate baclofen titration, her hypertonia and enteral pharmacologic regimen were both reduced. We demonstrate that ITB is a viable modality for treating refractory dystonia and spasticity secondary to GA1; it can objectively reduce hypertonia, subjectively improve quality of life, and minimize the side effect profile of otherwise extensive pharmacologic therapies.

## Introduction

Glutaric aciduria type I (GA1) is biochemically characterized by a deficiency in glutaryl-coenzyme A dehydrogenase and subsequent accumulation of putatively neurotoxic metabolites. Striatal injury, a neuropathologic hallmark, arises from a combination of prenatal dysgenesis, acute encephalopathic crises, and insidious damage; its clinical correlate is that of extrapyramidal symptoms, spasticity, and parkinsonism which can be life-limiting [1-4]. Although advances have been made to identify GA1 on newborn screening for early implementation of disease-modifying nutritional therapy, it remains that some sequelae are inevitable given that neurologic injury likely occurs in utero, dietary therapy is imperfect and not universally accessible, and that there is a paucity of evidence in support of any given regimen for symptomatic relief [1].

Because neurologic injury is largely unavoidable in GA1, dystonia and spasticity can be particularly problematic [5]. Unfortunately, first-line multimodal pharmacotherapy is often ineffective. Patients either suffer from inadequate motor relief or from the substantial side effect profiles of benzodiazepines, baclofen, and anticholinergics each of which are frequently used at high doses to combat tolerance. Intrathecal baclofen (ITB) and intraventricular baclofen (IVB) are both promising alternatives for severe cases of secondary dystonia as they offer titratable and substantially reduced doses of baclofen that is, unlike systemic therapy, delivered proximally to the site of baclofen’s anti-dystonic activity [6,7]. While IVB delivery has two early reports of use in GA1, the efficacy of ITB delivery has not been established [6]. Here we present the first reported treatment course of a patient with severe, medically refractory dystonia and spasticity secondary to GA1 that was effectively treated with ITB.

## Case presentation

The patient is a 15-year-old girl who was diagnosed with GA1 based on newborn screening. Despite early intervention with a low-lysine diet, carnitine supplementation, and emergency dietary modifications, she suffered from two encephalopathic crises in her lifetime and substantial neuromuscular sequelae. Most significantly, she had both fixed and mobile dystonia and spastic quadriplegic cerebral palsy without parkinsonism (Gross Motor Function Classification System level III-IV). Prior to neurosurgical evaluation, her movement disorder was treated with an extensive multimodal set of sedatives, anticonvulsants, anxiolytics, and antispasmodics. At the time of her presentation, this regimen was becoming subjectively less efficacious.

**Figure 1:**
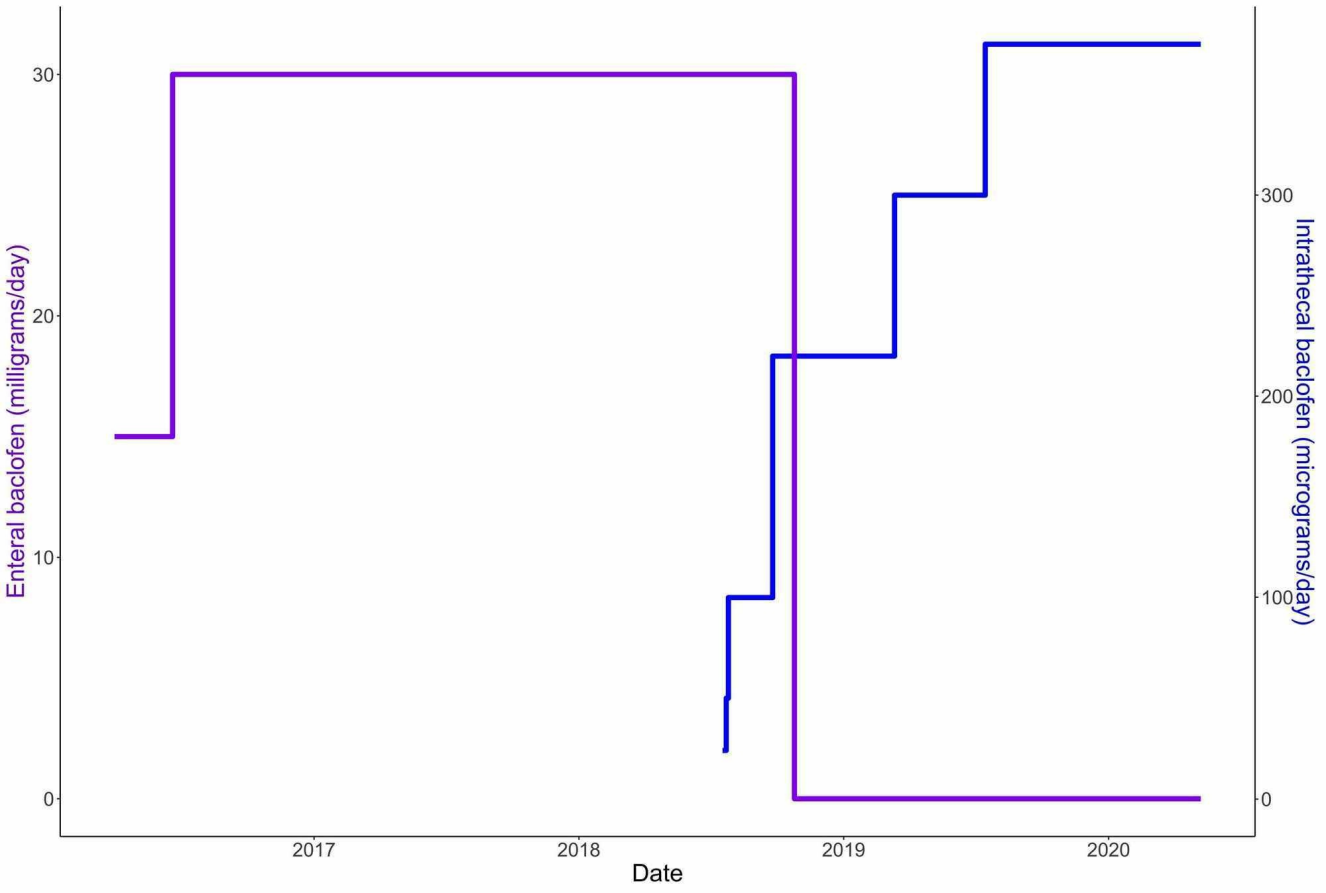
Enteral and intrathecal baclofen dosing relative to initiation and titration of the intrathecal baclofen Purple: enteral baclofen dosing (milligrams/day). Blue: intrathecal baclofen dosing (micrograms/day).

The patient underwent placement of an ITB pump, with the catheter tip at T1-T2 and an abdominal infusion pump (Medtronic SynchroMed II, Medtronic Inc., Dublin, Ireland) in a standard fashion under general anesthesia. She had no perioperative complications, and her ITB dose was titrated to 375 µg daily (Figure 1). With this dose increase over time, she was able to be tapered from enteral baclofen and scheduled diazepam (Table 1). In the two years since initiation of intrathecal therapy, she has not required any injections or other procedures for dystonia management; this contrast significantly with the 1980 units of botulinum toxin she cumulatively received over five injections in the two years prior to intrathecal therapy.

**Table 1:** Preoperative and serial postoperative medical therapies and Barry-Albright Dystonia Scale scores

	Preoperative	Postoperative			
		1 week	3 months	1 year	2 years
Intrathecal baclofen (µg/day)	-	100	220	375	375
Enteral medications (mg/day)					
Baclofen	30	30	0	0	0
Clonazepam	0.6	0.6	0.6	0.6	0.6
Diazepam	1.25	prn	prn	prn	prn
Total Barry-Albright Dystonia Scale	12	9	9	NR	NR

Most importantly, the patient and family received significant clinical benefits from the intrathecal therapy aside from adjustments to her medication regimen. Standardized measurement of dystonia with the Barry-Albright Dystonia Scale showed reduced global dystonic movements (Table 1). Subjectively, the patient’s family reported improved function, most noted in the use of her wheelchair and daily activities, that has continued through two years of postoperative follow-up.

## Discussion

GA1 is characterized by abnormal perinatal neurologic development, encephalopathic crises precipitated by catabolic and physiologically stressful states, and insidious neurotoxicity resulting in substantial motor deficits. Although the neurologic manifestations of GA1 are highly variable - correlating poorly with enzymatic deficiency and altered metabolism, pathologic neuroimaging, and even amongst siblings - dystonia (and less frequently spasticity) can be severely disabling [2,3,8]. Unfortunately, there are no well-designed trials to demonstrate the efficacy of any medical therapy for dystonia secondary to GA1; however, the preponderance of data currently supports baclofen and benzodiazepines either as monotherapies or together for first-line symptom reduction. Even when combined with adjuvant therapies such as anticholinergics and botulism toxin injections, these regimens often provide inadequate relief and can be associated with tolerance and substantial side effects.

Intrathecal and intraventricular delivery of baclofen has been promising for many secondary causes of dystonia; by delivering baclofen proximally to its molecular targets, therapeutic doses can be orders of magnitudes lower than their enteral counterparts [6,7]. This theoretically reduces the risk of tolerance and dose-dependent side effects. To date, there have been two reported cases of IVB for dystonia secondary to GA1 with promising results [6]. These authors favor IVB to ITB for dystonia in GA1 given the equipoise in safety and that the mechanism of baclofen’s anti-dystonic (but not anti-spasmodic) action is presumed to be supraspinal. Two additional patients are referenced to have had treatment with ITB pumps, though lack of reported clinical details precludes meaningful comparison [5].

We demonstrated that ITB can be a viable modality for medically refractory dystonia secondary to GA1, especially in patients with concomitant spasticity. Although baclofen likely operates at a supraspinal level to ameliorate dystonia, ITB in this patient was easily titrated to an efficacious concentration well below what the patient required enterally and below previously reported values for generalized dystonia [7,9]. Further, the intrathecal thoracic catheter tip allows simultaneous high concentrations of baclofen proximal to its antispasmodic target. Taken together, we demonstrated that ITB, much like IVB, is an appropriate modality for dystonia and spasticity secondary to GA1 that is refractory to medical therapy. More research is needed to directly compare the efficacy of IVB and ITB in patients with secondary dystonia, including those with GA1.

## Conclusions

GA1, despite advances in early detection and preventive therapies, continues to present with problematic, medically refractory dystonia and, less frequently, spasticity. Here we report the treatment course of a patient with severe dystonia and spasticity secondary to GA1, demonstrating ITB as an efficacious option for neuromuscular palliation in this population.
